# Oxygen, Carbon, Nitrogen, Hydrogen, Phosphorus and Sulphur Changed by Adam’s Sin. (?)

**Published:** 1869-03

**Authors:** 


### Oxygen, Carbon, Nitrogen, Hydrogen, Phosphorus and Sulphur changed by Adam's sin. (?)

Prof. Charles W. Chancellor, M. D., of the Washington University, Baltimore, in his charge to the graduates, says:

"You have learned that in Eden all was perfect—the mineral, the vegetable, the animal, until 'man's first disobedience brought death into the world and all our woes.' That a violation of God's holy law not only changed man's moral nature, but the atoms of the whole world became impressed with the wicked influences of the spirit of evil. It made oxygen a destroyer, and fired it with a species of fury to seize upon and destroy its fellow atoms. It made nitrogen fickle, false and unreliable. It so changed the kingly elements of carbon, hydrogen, phosphorus and sulphur in their nature, that they yielded an unwilling and uncertain obedience to the operations of the great forces of the universe. They have no regard for the value or beauty of animal or vegetable life, but are only too happy when the process of disintegration sends them back to revel in the inorganic world of atoms.

The varied forms of disease which afflict mankind are but a tithe of all we may see, resulting from a prevalent want of harmony in the universe; and it is your province, gentlemen, as philosophical enquirers, to take this extended and just view of the original cause and widespread results of evil in the world."

If we only knew what were the properties of these elements previous to Adam, we should be able to judge better about the change. We had heard that Adam's sin inoculated the race with evil, but did not know that it had changed the natural elements. We are willing to accept almost any proposition concerning it, since Adam cannot be greatly injured by any accusation now brought against the purity of his life. Any one who desires to defend Mr. Adam, can put in a plea that he was probably ignorant of chemistry, and did not mistrust that slight changes would produce such fearful and fatal results.

